# Clinical characteristics and mutation spectrum in 33 Chinese families with familial exudative vitreoretinopathy

**DOI:** 10.1080/07853890.2022.2146744

**Published:** 2022-11-21

**Authors:** Jianbo Mao, Yijing Chen, Yuyan Fang, Yirun Shao, Ziyi Xiang, Hanxiao Li, Shixin Zhao, Yiqi Chen, Lijun Shen

**Affiliations:** a Department of Ophthalmology, Center for Rehabilitation Medicine, Affiliated People’s Hospital, Hangzhou Medical College, Hangzhou, PR China; b Department of Retina Center, Affiliated Eye Hospital of Wenzhou Medical University, Hangzhou, PR China; c Hangzhou TCM Hospital Affiliated to Zhejiang Chinese Medical University, Hangzhou, PR China

**Keywords:** LRP5, FZD4, TSPAN12, NDP, KIF11, foveal hypoplasia, familial exudative vitreoretinopathy

## Abstract

**Objective:**

To explore the clinical manifestations and search for the variants of six related genes (*LRP5*, *FZD4*, *TSPAN12*, *NDP*, *KIF11* and *ZNF408*) in Chinese patients with familial exudative vitreoretinopathy (FEVR), and investigate the correlation between the genetic variants and the clinical characteristics.

**Patients and methods:**

Clinical data, including the retinal artery angle, acquired from wide-field fundus imaging, structural and microvascular features of the retina obtained from optical coherence tomography (OCT) and OCT angiography (OCTA) were collected from 33 pedigrees. Furthermore, mutation screening was performed. Variants filtering, bioinformatics analysis and Sanger sequencing were conducted to verify the variants.

**Results:**

Twenty-one variants were successfully detected in 16 of 33 families, of which 10 variants were newly identified. The proportion of variants in *LRP5*, *FZD4*, *TSPAN12*, *NDP* and *KIF11* was 38.1% (8/21), 33.3% (7/21), 19.1% (4/21), 4.8% (1/21) and 4.8% (1/21), respectively. Three new variants were considered to be pathogenic or likely pathogenic. The FEVR group tended to exhibit a smaller retinal artery angle, higher incidence of foveal hypoplasia and lower vascular density compared to the control group. Patients who harboured variants of *FZD4* exhibited greater severity of FEVR than those with *LRP5* variants. However, those who harboured *LRP5* variants tended to possess lower foveal vascular density.

**Conclusions:**

Six known pathogenic genes were screened in 33 pedigrees with FEVR in our study, which revealed 10 novel variants. These findings enrich the clinical features and mutation spectrum in Chinese patients with FEVR, revealing the genotype-phenotype relationship, and contributing to the diagnosis and treatment of the disease.

**Key messages**
- We identified 21 variants in 5 genes (*LRP5, FZD4, TSPAN12, NDP* and *KIF11)* associated with FEVR, 10 of which are novel (three were pathogenic or likely pathogenic).
- The proportion of variants was the highest for the *LRP5* gene.
- *FZD4* variants may be responsible for greater FEVR severity than *LRP5* variants.

## Introduction

Familial exudative vitreoretinopathy (FEVR) is a rare inheritable ocular disorder characterized by abnormal retinal vascular growth. It usually occurs in full-term infants and children and can lead to fibrovascular proliferation, vitreoretinal traction, retinal folds and retinal detachment [1]. The clinical manifestations of FEVR vary considerably from no distinct symptoms to vision loss, presenting difficulties for clinical diagnosis and treatment. Fluorescein fundus angiography (FFA) remains the current gold standard for diagnosis; however, it is an invasive and extremely consuming procedure. Currently, wide-field fundus imaging is commonly used because it compensates for the shortcomings of FFA, facilitating 220–240° imaging of the retina. Optical coherence tomography (OCT) and OCT angiography (OCTA) are used to assess and quantify the structural and functional features of the retinal and choroidal vascular morphology, especially in the macula. These new modalities have found widespread application for diagnosing and monitoring the progression of FEVR.

The genetic causes of FEVR are numerous. The most common aetiology can be attributed to an autosomal dominant inheritance, but may also include autosomal recessive and X-linked recessive inheritance [2,3]. Currently, 15 genes and one locus are known to be associated with FEVR. Of these, 11 genes, including tetraspanin-12 (*TSPAN12*), zinc finger protein 408 (*ZNF408*), catenin alpha 1 (*CTNNA1*), catenin delta 1 (*CTNND 1*), atonal homolog 7 (*ATOH7*), RCC1 and BTB domain-containing protein 1 (*RCBTB1*), integrin-linked kinase (*ILK*), jagged canonical Notch ligand 1 (*JAG1*), discs large MAGUK scaffold protein 1 (*DLG1*), low-density-lipoprotein receptor-related protein 6 (LRP 6) and exudative vitreoretinopathy 3 (*EVR3*) on chromosome 11p12-13 [4–14], are non-syndromic. The remaining genes are associated with systemic diseases. The proteins encoded by low-density-lipoprotein receptor-related protein 5 (*LRP5*) are associated with osteoporosis-pseudoglioma syndrome [15], and the Norrie disease protein (*NDP*) is associated with Norrie disease [16]. The protein encoded by kinesin family member 11 (*KIF11*) [17] can cause autosomal dominant microcephaly with or without chorioretinopathy, lymphedema or intellectual disability. Catenin beta 1 (*CTNNB1*) is also an uncommon cause of microcephaly [18]. Frizzled-4 (*FZD4*) variants can result in hearing deficits and developmental delays [19]. Current research indicates that only around 40–50% of cases of FEVR harbour identifiable genetic variants [20–22], while the relationship between the genotype and clinical manifestations is complex, which are responsible for the challenges in early clinical diagnosis and effective treatment.

In this study, we investigated the genotype and clinical features of FEVR in members of families with and without this disease and compared the results to a healthy control group without any history of FEVR. We collected data using wide-field fundus imaging, OCT and OCTA to analyse the relationship between the phenotypes and genotypes of FEVR.

## Patients and methods

### Study participants

This study adhered to the Declaration of Helsinki and its protocol was approved by the ethics review board of Wenzhou Medical University Affiliated Eye Hospital. Informed consent was obtained from all participants involved in the study.

We enrolled probands and families associated with FEVR who visited Wenzhou Medical University Affiliated Eye Hospital between September 2017 and November 2021, in addition to the healthy members of the affected families. Normal individuals without an FEVR-related genetic family history or any history of retinal vascular diseases including hypertension, vein occlusions and diabetic retinopathy were enrolled as the control group. All individuals were native Chinese, without any history of premature birth or oxygen inhalation. The patients and controls were matched for age and gender.

### Clinical information collection

Basic demographic and medical information was collected from each family and comprehensive ophthalmic examinations were conducted for each of the probands and their family members at Wenzhou Medical University Affiliated Eye Hospital. The examinations included intraocular pressure measurement, visual acuity measurement, slit-lamp biomicroscopy, wide-field fundus imaging, OCT, OCTA and FFA, which were performed by the same qualified technician.

Patients were considered to have FEVR on the basis of ophthalmic testing if they exhibited at least one of the following typical clinical findings: (1) peripheral retinal avascularity, (2) severe subretinal exudation, (3) neovascularization, (4) retinal fold or detachment, (5) supraretinal peripheral fibrovascular mass, (6) macular ectopia or (7) vitreous haemorrhage. The examinations of family members can support the diagnosis, and subsequently, family pedigrees were drawn. The severity of each case of FEVR was further assessed and classified according to the grading system devised by Pendergast and Trese [23]. FEVR stages 1 and 2 were denoted as the mild phenotype and stages 3–5 were designated as the severe phenotype.

Wide-field fundus imaging was conducted using the Optos 200Tx (Optos, Marlborough, MA). We measured the retinal artery angle in the participators’ eyes in accordance with the Yugami correlated angle (YCA) defined by Nagura et al. [24]. First, the distance between the optic nerve head (ONH) and the fovea was measured. Subsequently, we drew a circle centred on the ONH with a radius of half the distance between the ONH and the fovea based on calculations performed using the ImageJ software. The points of intersection between the circle and the arteries of the upper and lower arcade were obtained, and the angle enclosed by the two lines joining the intersection points and the ONH was denoted as the retinal artery angle in the total study population. OCTA was performed using the RTVue XR Avanti AngioVue (Optovue Inc., Fremont, CA) and 3 × 3 mm scans centred on the fovea were obtained for each eye. Spectral domain OCT (Heidelberg Engineering, Heidelberg, Germany) was also performed. The images acquired *via* the conventional mode were used for structural grading of the fovea, which was classified as the presence or absence of foveal hypoplasia, as described by Thomas et al. [25]. Foveal hypoplasia was characterized by the presence of inner retinal layers in the fovea on spectral domain OCT. Inner retinal thickness (IRT) was defined as the distance between the internal limiting membrane (ILM) and outer border of the inner nuclear layer. Central macular thickness (CMT) was defined as the average thickness of the circle measuring 1 mm in diameter centred on the fovea. The IRT of the fovea and CMT were obtained to quantify the degree of foveal hypoplasia.

### Mutation screening and analysis

Peripheral blood samples were collected from the individuals and preserved at −80 °C before use. Whole-exome sequencing (Peking Tsingke Biotechnology Co., LTD, Peking, China) was performed for the probands who were diagnosed with FEVR and their families. Raw data were filtered using the quality control process. The Genome Analysis Toolkit (https://www.broadinstitute.org/gatk/) was to detect single nucleotide polymorphisms and insertion-deletion in the six known FEVR-associated genes (*LRP5*, *FZD4*, *TSPAN12*, *NDP*, *KIF11* and *ZNF408*). Finally, SnpEff (http://snpeff.sourceforge.net/SnpEff_manual.html) was used to annotate the information.

The 1000 Genome database (http://www.1000genomes.org/), ExAC (http://exac.broadinstitute.org/), Exome Sequencing Project (https://evs.gs.washington.edu/EVS/) and National Centre for Biotechnology Information (https://www.ncbi.nlm.nih.gov/) were searched for each variant to determine if it had been reported previously. Variants that were not found in these databases were considered novel. The potential deleterious effect of each variant was assessed using nine variant bioinformatics tools including SIFT, Poly-phen2HVAR, LRT, Mutation Taster, FATHMM, CADD, GERP++, phyloP100way_vertebrate, and SiPhy_29way_logOdds. Amino acid sequences corresponding to the alterations in the genotype changes were obtained from the National Centre for Biotechnology Information (http://www.ncbi.nlm.nih.gov/) and the European Bioinformatics Institute (https://www.ebi.ac.uk/Tools/msa/tcoffee/).

Furthermore, Sanger sequencing and co-segregation detection were performed for the 33 pedigrees. Finally, the pathogenicity of these variants was evaluated according to the standards and guidelines provided by the American College of Medical Genetics and Genomics (ACMG).

### Data analysis

The clinical phenotype of the patients was graded and classified [23]. All detected variants were classified according to the type and the number of variants. We further explored the characteristics of the clinical and genomic variations as well as the potential genotypic–phenotypic correlations.

The Statistical Package for the Social Sciences version 23 (SPSS Inc., Chicago, IL) was used for statistical analysis. Normality of distribution of the variables was inspected using the Shapiro–Wilk test. The Student’s *t*-test was used to compare data with normal distributions. Non-normally distributed data of the patients and controls were compared using the Mann–Whitney U test. Nonparametric data were compared using the Kruskal–Wallis H test and chi-squared test. *p* Values less than 0.05 were considered statistically significant.

## Results

In this study, the diagnosis and grading of FEVR were performed after a comprehensive clinical analysis based on the results of FFA and genetic screening of 33 families. This study enrolled 127 participants from 33 pedigrees, including 81 patients with FEVR and 46 healthy individuals (Table 1). The FEVR group comprised 40 men and 41 women, whose average age was 22.0 ± 17.3 years old. Most patients presented with mild symptoms. The average age of the probands from the 33 FEVR families was 9.1 ± 11.7 years old, of which 17 were boys/men and 16 were girls/women. The majority of probands presented with a more severe phenotype, including foveal hypoplasia, compared to their family members. Variants were detected in 48 participants with FEVR, who underwent further phenotype and genotype analysis.

**Table 1.** Clinical phenotype and genotype of participants.

Features		Proband	FEVR	Healthy family members	Normal group
		(*n* = 33)	(*n* = 81)	(*n* = 46)	(*n* = 49)
Age, mean (SD), y		9.1 ± 11.7	22.0 ± 17.3	37.2 ± 15.6	21.3 ± 12.6
					
Male/Female		17/16	40/41	22/24	20/29
					
Variants (numbers of individuals)	With	16	40	8	–
	*FZD4*	6	15	0	–
	*LRP5*	2	8	7	–
	*NDP*	1	1	1	–
	*FZD4*	3	8	0	–
	*KIF11*	1	1	0	–
	*FZD4 + LRP5*	1	4	0	–
	*LRP5 + FZD4*	1	2	0	–
	*LRP5 + LRP5*	1	1	0	–
	Without	17	41	38	–
					
Ocular symptoms (numbers of individuals)	Retinal detachment	10	16	–	–
	Retinal breaks	5	6	–	–
	High intraocular pressure	5	6	–	–
	Cataract	5	8	–	–
	Strabismus	10	12	–	–
	Nystagmus	5	9	–	–
					
Stages of FEVR (numbers of eyes)	Mild	52	137	–	–
	1	32	100	–	–
	2	20	37	–	–
	Severe	14	25	–	–
	3	8	8	–	–
	4	4	11	–	–
	5	2	6	–	–
Foveal Hypoplasia (numbers of eyes)	With	31	48	–	–
	Without	19	60	–	–
				
Previous treatment (numbers of eyes)	None	34	107	–	–
	Laser/Anti-VEGF	21	39	–	–
	Incisional surgery	11	16	–	–

### Number of pedigree variants

**Figure 1.**
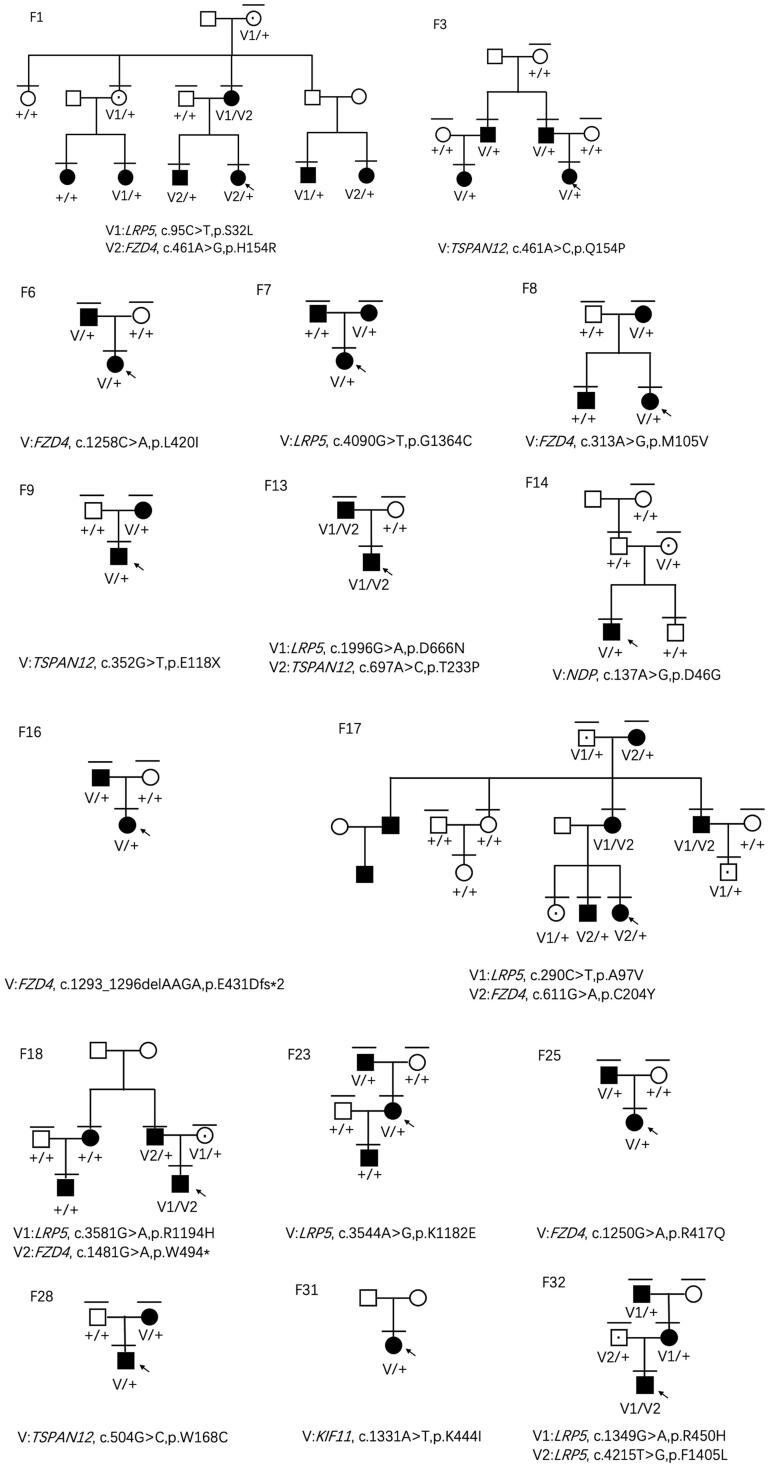
The 16 family pedigrees with mutations. The individuals enrolled in our study are marked by the horizontal line above the figures. The probands are pointed by the black arrow. The square means male and the circle means female. The figures filled with black refer to FEVR patients and blank figures refer to healthy family members. The healthy carriers are represented by a black dot in the blank figure. F represents a family. V represents a variant, and + indicates a normal allele. V1 and V2 are used to discriminate two genotypes when FEVR patients carry digenic variants.

Twenty-one variants were detected in the 33 FEVR families, of which 10 had not been previously reported and are hence considered as novel (Table 2). Variants were detected in only in 16 of the 33 families. Analysis of the family pedigrees (Figure 1) revealed more than one in three participants carried genetic variants, including 40 patients with FEVR and 8 carriers. A total of 41 patients had single-site variants and 7 patients had variants in two sites, including 6 carrying two types of gene variants and 1 harbouring two variants in *LRP5* (Table 1).

**Table 2.** Twenty-one variants in *LRP5*, *FZD4*, *TSPAN12*, *NDP* and *KIF11.*

Gene	Family	Type	Variant	Allele frequency in			Reference	rs ID	Heredity	ACMG
				ESP 6500	1000G	ExAC				
*LRP5*	F1	Missense	c.95C > T,p.S32L	0	0	<0.01%	Reported	rs755388709	Maternal	Under certain significance
*LRP5*	F7	Missense	c.4090G > T,p.G1364C	0	0	0	Novel	–	Maternal	Under certain significance
*LRP5*	F13	Missense	c.1996G > A,p.D666N	0	0.0008	0.0002	Reported	rs180941579	Paternal	Likely benign
*LRP5*	F17	Missense	c.290C > T,p.A97V	0	0.0008	0	Reported	rs143433231	Maternal	Likely benign
*LRP5*	F18	Missense	c.3581G > A,p.R1194H	0	0.0002	0	Reported	rs201017887	Maternal	Under certain significance
*LRP5*	F23	Missense	c.3544A > G,p.K1182E	0	0	0	Reported	rs950665645	Paternal	Under certain significance
*LRP5*	F32	Missense	c.1349G > A,p.R450H	0	0	<0.01%	Reported	rs1335197449	Maternal	Under certain significance
*LRP5*	F32	Missense	c.4215T > G,p.F1405L	0	0	0	Novel	–	Paternal	Under certain significance
*FZD4*	F1	Missense	c.461A > G,p.H154R	0	0	0	Reported	rs1334686841	Maternal	Unde rcertain significance
*FZD4*	F6	Missense	c.1258C > A,p.L420I	0	0	0	Novel	–	Paternal	Under certain significance
*FZD4*	F8	Missense	c.313A > G,p.M105V	0	0	<0.01%	Reported	rs80358284	Maternal	Pathogenic
*FZD4*	F16	Frameshift	c.1293_1296delAAGA,p. E431Dfs*2	0	0	0	Novel	–	Paternal	Likely pathogenic
*FZD4*	F17	Missense	c.611G > A,p.C204Y	0	0	0	Reported	rs1064794064	Maternal	Pathogenic
*FZD4*	F18	Nonsense	c.1481G > A,p.W494*	0	0	0	Novel	–	Paternal	Pathogenic
*FZD4*	F25	Missense	c.1250G > A,p.R417Q	0	0	0	Reported	rs80358294	Paternal	Pathogenic Pathogenic
*TSPAN12*	F3	Missense	c.461A > C,p.Q154P	0	0	0	Novel	–	Paternal	Under certain significance
*TSPAN12*	F9	Nonsense	c.352G > T,p.E118X	0	0	0	Novel	–	Maternal	Pathogenic
*TSPAN12*	F13	Missense	c.697A > C,p.T233P	0	0	0	Novel	–	Paternal	Under certain significance
*TSPAN12*	F28	Missense	c.504G > C,p.W168C	0	0	0	Novel	–	Maternal	Under certain significance
*NDP*	F14	Missense	c.137A > G,p.D46G	0	0	0	Novel	–	Maternal	Under certain significance
*KIF11*	F31	Missense	c.1331A > T,p.K444I	0	0	0	Reported	rs759757759	De novo	Under certain significance

**Figure 2.**
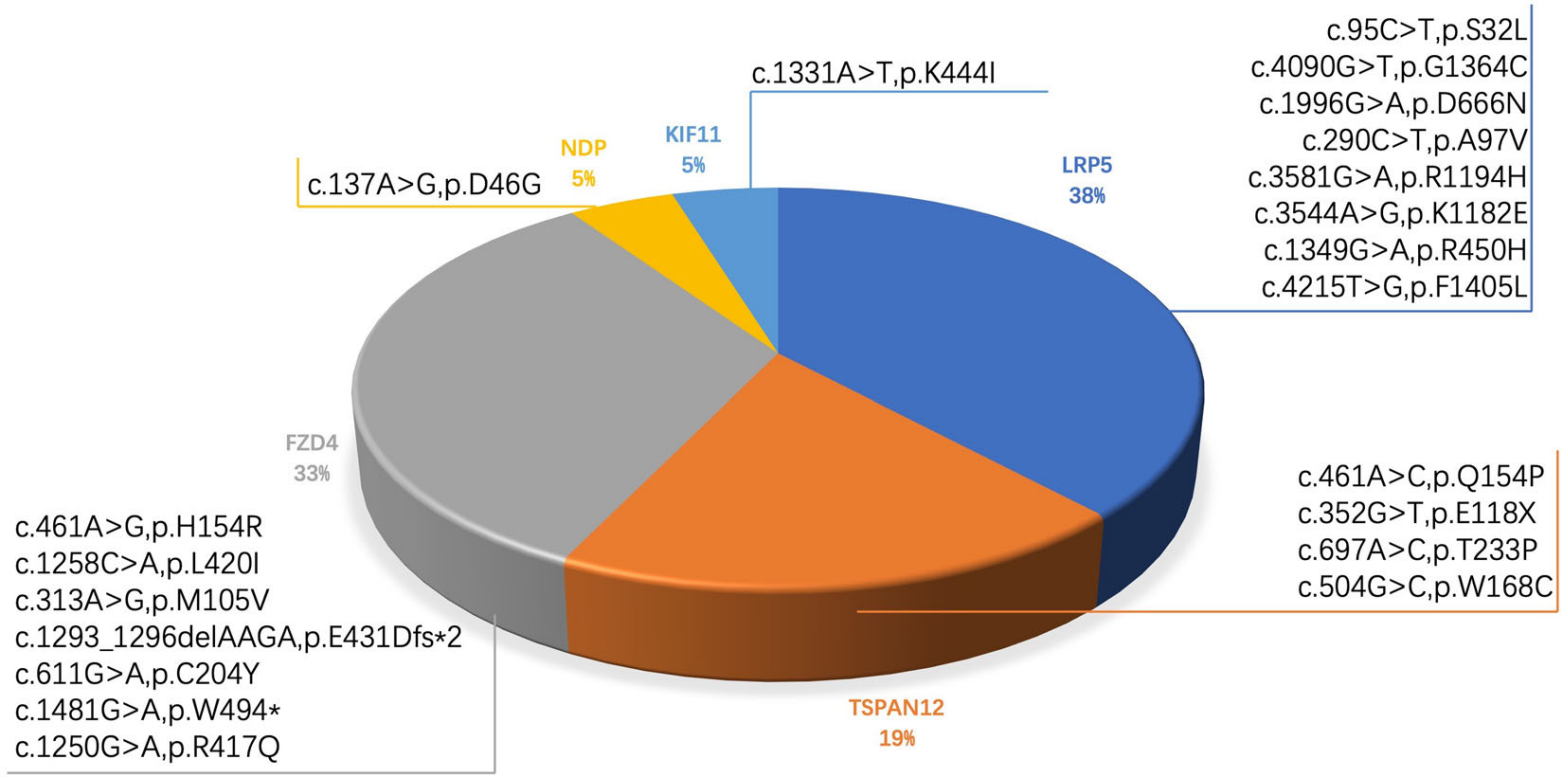
The mutation spectrum in the Chinese FEVR cohort. Each coloured box represents the different related genes, showing the proportion of different genes.

Twenty-one variants in five (*LRP5*, *FZD4*, *TSPAN12*, *NDP* and *KIF11*) of the six genes associated with FEVR were detected in the 33 families, including 81 patients with FEVR and 41 healthy family members, which accounted for the clinical symptoms of 16 families (16/33, 48.5%). As shown in Figure 2, variants in the *LRP5* gene constituted the highest proportion (8/21, 38.1%) of variants, followed by variants in *FZD4* (7/21, 33.3%) and *TSPAN12* (4/21, 19.1%), respectively. Only one variant was present in *NDP* and *KIF11* (1/21, 4.8% each), and no pathogenic variants were identified in *ZNF408*.

**Figure 3.**
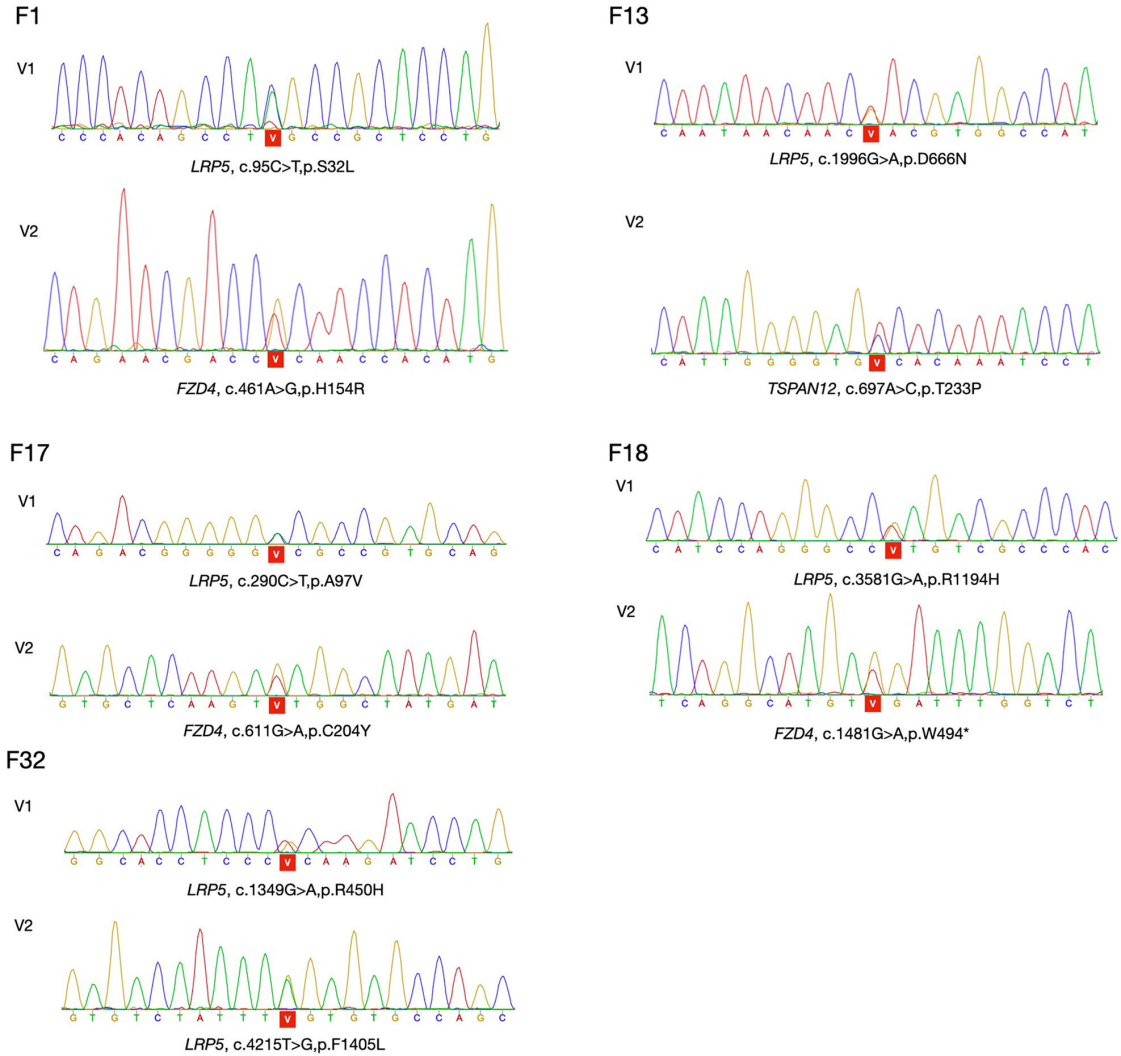
The chromatograms of sequencing results in five families carrying two variants. F represents a family, and V represents a variant.

The distribution of the 10 newly identified variants was as follows: 2 in *LRP5* (family 7 and family 32), 3 in *FZD4* (family 6, family 16 and family 18), 4 in *TSPAN12* (family 3, family 9, family 13 and family 28) and 1 in *NDP* (family 14). Of the 11 previously reported variants, 6 were identified in *LRP5* (family 1, family 13, family 17, family 18, family 23 and family 32), 4 in *FZD4* (family 1, family 8, family 17 and family 25), 1 in *KIF11* (family 31) (Table 2). Two variants were discovered in five families (family 1, family 13, family 17, family 18 and family 32), respectively. The chromatograms of their sequencing results are depicted in Figure 3.

The variants in *TSPAN12* (c.352G > T, p.E118X) and *FZD4* (c.1481G > A, p.W494*) were characterized as nonsense variants. A frameshift variant was identified in *FZD4* (c.1293_1296delAAGA;p.E431Dfs*2), while the remaining 18 were missense variants. Except for *NDP* (c.137A > G,p.D46G) and *KIF11* (c.1331A > T,p.L444I), the variants of the other three genes were co-segregated with the disease in an autosomal dominant manner.

The pathogenicity of the newly identified variants was further verified using nine bioinformatics tools and amino acid sequence comparisons. According to the guidelines provided by the ACMG, the variants in *TSPAN12* (c.352G>T, p.E118X) and *FZD4* (c.1481G > A, p.W494*) were perceived as pathogenic. *FZD4* (c.1293_1296delAAGA, p.E431Dfs*2) was predicted to be likely pathogenic. The proband of family 9 with the *TSPAN12* (c.352G>T, p.E118X) variant, had retinal neovascularization in one eye (and underwent laser treatment), while the other eye was only mildly affected and considered as having stage 1 disease. Peripheral avascular zones were detected in both eyes of the mother. In family 18, the proband and his father harboured an *FZD4* (c.1481G > A, p.W494*) variant, and underwent laser treatment for retinal breaks. In family 16, *FZD4* (c.1293_1296delAAGA,p.E431Dfs*2) was detected, and the proband developed retinal detachment in one eye and underwent surgical repair, while the contralateral eye was only mildly affected. Her father had peripheral avascular zones in both eyes.

### Clinical manifestations

**Figure 4.**
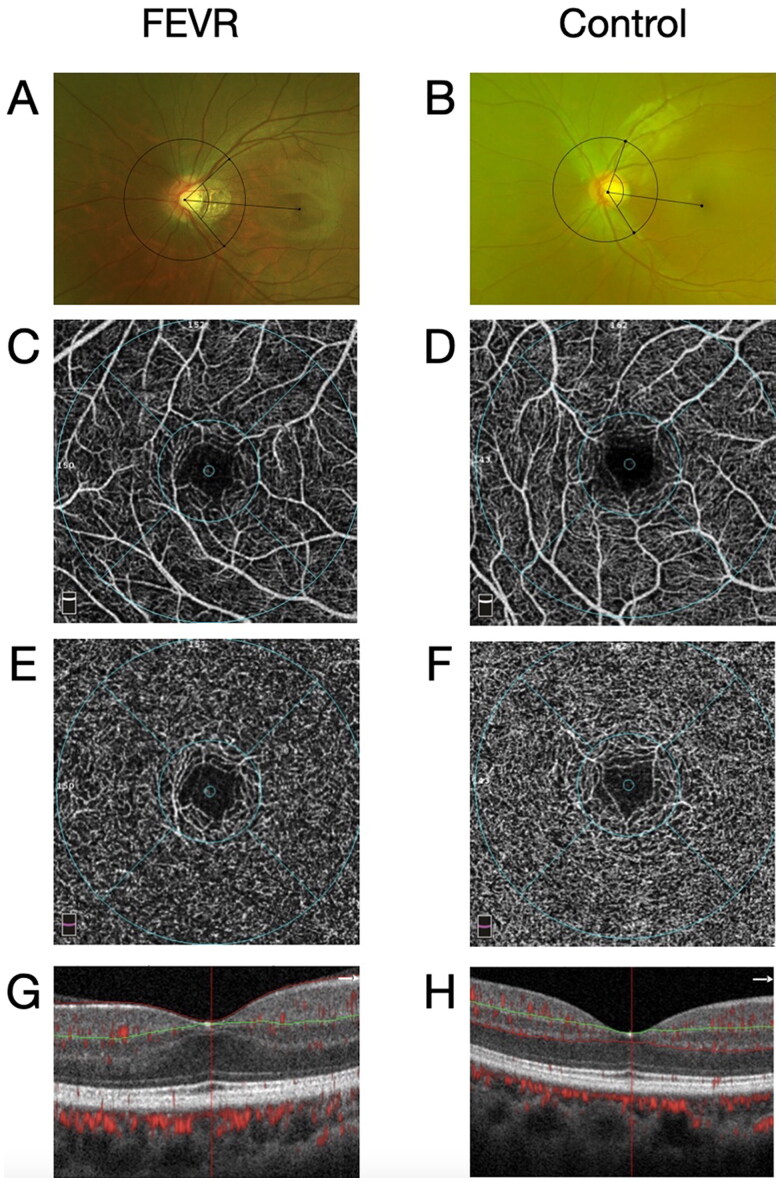
Retinal artery angle, OCTA parameters and incidence of foveal hypoplasia in eyes with FEVR and normal eyes. The FEVR patients had a smaller retinal artery angle (A) than the normal individuals (B). The vascular density of SCP and DCP decreased in the eyes of FEVR patients (C, E) when compared to the normal eyes (D, F). The preserved foveal inner retinal layer was noted in the patients affected by FEVR (G) while the normal individuals had a normal structure (H).

We compared the results of wide-field fundus imaging, OCT and OCTA between the FEVR and control groups (Table 3 and Figure 4). Patients with FEVR tended to have a smaller retinal artery angle on wide-field fundus imaging compared to the controls (*p*<0.001). The incidence of foveal hypoplasia was higher in patients with FEVR than that in the controls (*p* <0.001). The foveal IRT was significantly higher in eyes with FEVR (*p* < 0.001). OCTA-acquired vessel densities of the superficial capillary plexus (SCP) and deep capillary plexus (DCP) from the whole retinal image and the parafovea decreased in FEVR (P_SCP whole image _= 0.020, P_SCP parafovea _= 0.009, P_DCP whole image _<0.001, P_DCP parafovea _<0.001). Additionally, the foveal vessel density in a 300-µm wide region around the foveal avascular zone (FAZ) (FD-300) was lower in the FEVR group (*p* = 0.001). However, the acircularity index (AI) of the FAZ was greater in eyes with FEVR (*p* < 0.001). As shown in Table 4, the retinal artery angle was smaller in FEVR eyes with foveal hypoplasia compared to eyes without foveal hypoplasia (*p* = 0.013). The CMT and IRT of the fovea were significantly higher in eyes with foveal hypoplasia. Moreover, FEVR eyes with foveal hypoplasia exhibited a smaller FAZ area and perimeter, higher AI and lower FD-300. The vascular densities of the DCP on the whole and parafoveal images, and SCP on the parafoveal image were lower, while those of the foveal SCP and DCP were higher in eyes with foveal hypoplasia. The IRT was negatively correlated with the FAZ area (*r*= −0.745, *p* < 0.001) and perimeter (*r*= −0.731, *p* < 0.001), while the IRT was positively correlated with the vascular density of the SCP (*r* = 0.501, *p* = 0.017) and DCP (*r* = 0.578, *p* = 0.005) in the fovea. The retinal parameters at different FEVR stages were further analyzed and compared. Foveal hypoplasia was observed in all patients with severe disease compared to patients with mild FEVR. Further statistical analyses were performed for eyes in stages 1 and 2, owing to the small sample size of the severe stages, i.e. stages 3–5. The CMT was higher in stage 2 compared to stage 1 (*p* = 0.014). The foveal vascular density of the SCP was higher in eyes designated as stage 2 (*p* = 0.019).

**Table 3.** The clinical characteristics of eyes with FEVR and control eyes.

Parameters			FEVR(95%CI)	Control(95%CI)	*p* Value
Retinal artery angle	*N*		124	76	–
	Age (years)		23.3 ± 16.4	21.3 ± 12.6	0.522
	Angle (°)		90.30 ± 19.46	122.10 ± 13.34	<0.001*
			(86.85–93.76)	(119.06–125.15)	
					
Macular structure	*N*		108	76	–
	Age (years)		21.9 ± 17.8	21.3 ± 12.6	0.840
	With FH/All (%)		48/108(44.4%)	3/76(3.9%)	<0.001*
	CMT		259.54 ± 56.66	248.41 ± 15.31	0.399
			(247.99–271.08)	(244.91–251.91)	
	IRT		12.47 ± 21.70	0.78 ± 4.09	<0.001*
			(7.30–16.03)	(0.16–1.71)	
					
OCTA	*N*		70	76	–
	Age (years)		25.6 ± 18.8	21.3 ± 12.6	0.334
	FAZ	Area (mm^2^)	0.27 ± 0.13	0.27 ± 0.09	0.724
			(0.24–0.30)	(0.25–0.29)	
		Perimeter (mm)	2.08 ± 0.55	2.06 ± 0.38	0.782
			(1.95–2.21)	(1.97–2.15)	
		AI	1.18 ± 0.14	1.13 ± 0.04	<0.001*
			(1.15-1.21)	(1.12-1.14）	
		FD-300(%)	48.50 ± 6.74	51.42 ± 3.82	0.001*
			(46.87–50.13)	(50.55–52.29)	
	Whole image VD (%)	SCP	46.75 ± 4.58	48.17 ± 2.11	0.020*
			(45.65–47.84)	(47.69–48.65)	
		DCP	47.53 ± 5.44	52.51 ± 3.00	<0.001*
			(46.23–48.82)	(51.83–53.20)	
	Foveal VD (%)	SCP	22.66 ± 9.60	20.97 ± 5.49	0.721
			(20.37–24.95)	(19.72–22.23)	
		DCP	34.48 ± 8.61	35.38 ± 6.55	0.479
			(32.43–36.53)	(33.88–36.87)	
	Parafoveal VD (%)	SCP	49.12 ± 4.74	50.80 ± 2.52	0.009*
			(47.99–50.25)	(50.22–51.37)	
		DCP	49.39 ± 6.10	54.66 ± 3.16	<0.001*
			(47.94–50.84)	(53.94–55.38)	

*p < 0.05.

*N*: number of eyes; FH: foveal hypoplasia; CMT: centre macular thickness; IRT: inner retinal thickness; FAZ: foveal avascular zone; AI: acircularity index; FD-300: the foveal vessel density in a 300-μm-wide region around FAZ; VD: vascular density; SCP: superficial capillary plexus; DCP: deep capillary plexus.

**Table 4.** The clinical characteristics of different severity of FEVR.

Parameters			With FH	Without FH	P	Stage 1	Stage 2	*p*
Retinal artery angle	*N*		32	66		76	32	
	Angle (°)		81.11 ± 21.28	91.57 ± 17.46	0.013*	91.60 ± 18.24	89.95 ± 21.02	0.682
			(73.44–88.78)	(87.28–95.86)		(87.44–95.77)	(82.37–97.53)	
								
Macular structure	*N*		35	60		57	28	
	CMT		277.85 ± 89.85	249.33 ± 17.12	0.018*	248.84 ± 37.82	273.04 ± 78.54	0.014*
			(246.50–309.20)	(244.94–253.71)		(238.81–258.88)	(242.58–303.49)	
	IRT		36.17 ± 22.58	0 ± 0 (0–0)	<0.001*	8.82 ± 21.04	12.60 ± 18.64	0.112
			(27.74–44.60）			(3.24–14.41）	(5.64–19.56)	
								
OCTA	*N*		23	47		41	25	
	FAZ	Area (mm^2^)	0.22 ± 0.16	0.29 ± 0.11	0.006*	0.28 ± 0.13	0.25 ± 0.12	0.486
			(0.15–0.29)	(0.26–0.33)		(0.24–0.32)	(0.21–0.30)	
		Perimeter (mm)	1.84 ± 0.75	2.19 ± 0.40	0.031*	2.15 ± 0.54	1.99 ± 0.46	0.224
			(1.50–2.18)	(2.07–2.30)		(1.97–2.33)	(1.80–2.18)	
		AI	1.23 ± 0.20	1.16 ± 0.08	0.020*	1.20 ± 0.17	1.15 ± 0.05	0.629
			(1.14–1.33)	(1.13–1.18)		(1.14–1.25)	(1.13–1.17)	
		FD-300 (%)	46.04 ± 9.17	49.79 ± 4.12	0.006*	48.60 ± 4.94	50.06 ± 3.68	0.450
			(41.87–50.22)	(48.58–51.00)		(47.02–50.18)	(48.54–51.57)	
	Whole image VD (%)	SCP	46.07 ± 7.14	47.01 ± 2.54	0.940	46.31 ± 4.51	47.97 ± 2.40	0.416
			(42.98–49.16)	(46.26–47.75)		(44.89–47.74)	(46.98–48.96)	
		DCP	44.93 ± 6.07	48.75 ± 4.66	0.005*	47.89 ± 5.48	47.96 ± 4.48	0.781
			(47.38–50.12)	(47.38–50.12)		(46.16–49.62)	(46.11–49.81)	
	Foveal VD (%)	SCP	28.17 ± 13.35	19.90 ± 5.54	0.008*	21.10 ± 8.38	25.82 ± 9.34	0.019*
			(22.40–33.94)	(18.27–21.53)		(18.45–23.74)	(21.97–29.68)	
		DCP	37.66 ± 12.05	32.79 ± 7.51	0.005*	33.90 ± 8.69	36.03 ± 8.09	0.327
			(32.45–42.88)	(30.59–34.99)		(31.16–36.65)	(32.69–39.37)	
	Parafoveal VD (%)	SCP	47.67 ± 7.22	49.76 ± 2.62	0.008*	48.94 ± 4.92	49.98 ± 2.18	0.947
			(44.55–50.79)	(48.99–50.53)		(47.39–50.50)	(49.08–50.88)	
		DCP	46.20 ± 7.09	50.79 ± 4.96	0.042*	49.99 ± 6.00	49.30 ± 5.04	0.496
			(43.14–49.27)	(49.34–52.25)		(48.09–51.88)	(47.22–51.38)	

*p< 0.05.

*N*: number of eyes; FH: foveal hypoplasia; CMT: centre macular thickness; IRT: inner retinal thickness; FAZ: foveal avascular zone; AI: acircularity index; FD-300: the foveal vessel density in a 300-μm-wide region around FAZ; VD: vascular density; SCP: superficial capillary plexus; DCP: deep capillary plexus.

### Genotype–phenotype correlations

We included 39 participants with FEVR (78 eyes) who harboured variants from amongst the 33 families (Table 5) to investigate the relationship between the causative genes and clinical symptoms of FEVR. Participants with poor image quality were excluded. The parameters of patients with monogenic variants in *LRP5*, *FZD4* and *TSPAN12* were compared further due to the sample size. The phenotypic severity varied among the genotypes (P*_LRP5_*_-_ *_FZD4_*_-_*_TSPAN12 _*= 0.002) as follows. *FZD4* was associated with FEVR of greater severity than *LRP5* (P*_LRP5_*_-_*_FZD4 _*= 0.001). The frequency of foveal hypoplasia was the highest in patients with the *FZD4* variants; however, the frequency of hypoplasia did not differ significantly among the variants.

**Table 5.** The clinical characteristics of different genotypes in FEVR cohort.

Parameters			Monogenic variants (95%CI)	Digenic variants (95%CI)	*p* Value	*LRP5* (95%CI)	*FZD4* (95%CI)	*TSPAN12* (95%CI)	*p* Value
Stages of FEVR	*N*		66	12	–	16	30	16	–
	Mild	1	34	5	0.579	14	11	7	0.002*
		2	20	5		2	10	8	
	Severe	3	4	0		0	2	0	
		4	6	1		0	5	1	
		5	2	1		0	2	0	
									
Retinal artery Angle	*N*		50	8	–	15	18	13	–
	Angle (°)		86.37 ± 22.13	82.43 ± 26.11	0.650	81.82 ± 16.52	93.70 ± 17.64	88.22 ± 30.39	0.289
			(80.08–92.66)	(60.60–104.25)		(72.67–90.97)	(84.93–102.48)	(69.86–106.59)	
									
Macular Structure	With FH/All (%)		22/44(50.0%)	7/12(58.3%)	0.609	4/12(33.3%)	11/20(55.0%)	5/10(50.0%)	0.523
	CMT		265.87 ± 45.27	249.67 ± 28.42	0.229	265.00 ± 60.11	260.38 ± 37.08	268.70 ± 36.77	0.653
			(251.20–280.55)	(227.82–271.51)		(230.29–299.71)	(237.98–282.79)	(242.40–295.00)	
	IRT		10.00 ± 16.53	21.11 ± 26.27	0.386	5.09 ± 8.75	7.71 ± 15.15	11.60 ± 15.61	0.652
			(4.57 ± 15.43)	(0.92–41.30)		(0.78–10.97）	(1.03–16.46)	(0.43–22.77)	
OCTA	N		20	9	–	6	9	5	–
	FAZ	Area (mm^2^)	0.28 ± 0.13	0.27 ± 0.21	0.468	0.25 ± 0.06	0.36 ± 0.17	0.21 ± 0.03	0.113
			(0.22–0.35)	(0.11–0.43)		(0.18–0.32)	(0.22–0.50)	(0.17–0.25)	
		Perimeter (mm)	2.20 ± 0.50	1.95 ± 0.89	0.664	2.32 ± 0.55	2.34 ± 0.53	1.83 ± 0.18	0.175
			(1.96–2.44)	(1.27–2.64)		(1.74–2.89)	(1.90–2.78)	(1.62–2.05)	
		AI	1.19 ± 0.21	1.18 ± 0.10	0.809	1.33 ± 0.35	1.14 ± 0.02	1.12 ± 0.04	0.013*
			(1.09–1.29)	(1.10–1.25)		(0.96–1.70)	(1.12–1.15)	(1.07–1.17)	
		FD-300 (%)	47.72 ± 5.87	50.49 ± 4.01	0.438	42.66 ± 8.06	50.45 ± 2.90	49.43 ± 1.40	0.024*
			(44.89–50.55)	(47.41–53.57)		(34.20–51.12)	(48.02–52.87)	(47.70–51.17)	
	Whole image VD (%)	SCP	46.49 ± 4.37	49.82 ± 5.10	0.153	43.53 ± 6.08	48.00 ± 3.30	47.32 ± 1.90	0.191
			(44.45–48.53)	(45.90–53.75)		(37.16–49.91)	(45.46–50.54)	(44.97–49.67)	
		DCP	46.85 ± 5.39	41.37 ± 5.93	0.021*	46.02 ± 4.72	48.02 ± 6.42	45.74 ± 4.69	0.543
			(44.33–49.37)	(36.81–45.93)		(41.06–50.97)	(43.09–52.96)	(39.92–51.56)	
	Foveal VD (%)	SCP	21.92 ± 6.26	29.11 ± 15.40	0.208	17.62 ± 2.99	21.02 ± 6.55	28.68 ± 1.65	0.010*
			(18.98–24.85)	(17.28–40.95)		(14.48–20.76)	(15.99–26.06)	(26.64–30.72)	
		DCP	33.99 ± 8.78	37.30 ± 14.76	0.501	29.78 ± 3.48	30.27 ± 11.58	40.34 ± 2.10	0.034*
			(29.88–38.10)	(25.95–48.65)		(26.13–33.44)	(24.36–42.17)	(37.73–42.95)	
								
	Parafoveal VD (%)	SCP	48.56 ± 4.91	51.88 ± 4.45	0.062	45.63 ± 6.92	50.47 ± 3.75	48.64 ± 2.30	0.231
			(46.26–50.86)	(48.46–55.30)		(38.37–52.90)	(47.58–53.35)	(45.79–51.49)	
		DCP	48.44 ± 5.80	42.23 ± 7.57	0.022*	47.78 ± 4.82	49.79 ± 7.02	46.78 ± 4.87	0.399
			(45.72–51.15)	(36.41–48.05)		(42.73–52.84)	(44.39–55.19)	(40.73–52.83)	

*p < 0.05.

*N*: number of eyes; FH: foveal hypoplasia; CMT: centre macular thickness; IRT: inner retinal thickness; FAZ: foveal avascular zone; AI: acircularity index; FD-300: the foveal vessel density in a 300-μm-wide region around FAZ; VD: vascular density; SCP: superficial capillary plexus; DCP: deep capillary plexus.

The vessel densities of the DCP on the whole and parafoveal images were lower in participants with digenic variants (P_whole image _= 0.021; P_parafoveal _= 0.022) compared to those with monogenic variants. The AI of the FAZ in patients with *TSPAN12* was lower than that in patients with *LRP5* (P*_LRP5_*_-_*_FZD4_*_-_*_TSPAN12 _*= 0.013; P*_LRP5_*_-_*_TSPAN12 _*= 0.013). However, patients with FEVR who carried *LRP5* variants tended to have a lower foveal vascular density in the SCP and DCP than those harbouring *TSPAN12* variants (P_SCP[_*_LRP5_*_-_*_FZD4_*_-_*_TSPAN12_*_]_ = 0.010; P_SCP[_*_LRP5_*_-_*_TSPAN12_*_]_ = 0.010; P_DCP[_*_LRP5_*_-_*_FZD4_*_-_*_TSPAN12_*_]_ = 0.034; P_DCP[_*_LRP5_*_-_*_TSPAN12_*_]_ = 0.028). The FD-300 was also lower in patients with *LRP5* variants compared to those with *FZD4* variants (P *_LRP5_*_-_*_FZD4_*_-_*_TSPAN12_* = 0.024; P*_LRP5_*_-_*_FZD4_* = 0.028).

## Discussion

In this study, we employed comprehensive clinical screening and systematic analysis of six genes related to 33 pedigrees of families with FEVR. Ultimately, 21 gene variants of *FZD4*, *LRP5*, *NDP*, *TSPAN12* and *KIF11* were identified, which were found to be present in 49.4% (40/81) of patients with FEVR. Ten variants are novel since they have not been reported previously.

*LRP5,* which was present in 38.1% of cases in our study, was the most frequently occurring of the five genes with variants associated with FEVR, while *ZNF408* had no pathogenic variants, concordant with previous studies [21]. The gene mutation detection rates in FEVR seem to vary from study to study. Rao et al. [21] reported variants in *LRP5*, *FZD4*, *TSPAN12*, *NDP* and *KIF11* that accounted for 38.7% of patients with FEVR from 31 family pedigrees. Wang et al. [26] showed that up to 51.2% of the families had identifiable variants. It is possible that numerous uncharacterized pathogenic genes for FEVR remain undiscovered. *LRP5*, *FZD4*, *TSPAN12*, *NDP*, *LRP6* and *CTNNB1* have been identified as components of the Norrin/β-catenin signalling pathway. This pathway is highly conserved in biological evolution, playing a significant role in retinal angiogenesis. Recent studies have suggested that *RCBTB1, CTNND1, CTNNA1, ILK* and *DLG1* also participate in the regulation of the Norrin/β-catenin signalling pathway, resulting in abnormal growth of retinal blood vessels. Additionally, Notch ligand *JAG1* was reported to be a novel candidate gene for FEVR. These findings indicate that the pathogenesis of FEVR involves multiple signalling pathways.

The clinical manifestations of FEVR are complex and diverse [27]. We investigated and compared the structural and vessel-related parameters of the retina using wide-angle fundus imaging, OCT and OCTA and found that the retinal artery angle was smaller in eyes with FEVR compared to the control eyes. We speculated that retinal contraction in FEVR may be responsible for this change. Lee et al. [28] used hand-held OCT to investigate the vitreoretinal pathologies in FEVR. These pathologies included temporal and anterior displacement of the retina, significant vitreous capillary adhesion or traction and thickening of the retinal nerve fibre layer at the edge of ONH with protrusion of the adjacent retina. Nagura et al. [24] reported that the YCA was smaller in the eyes with an epiretinal membranes than that in the contralateral eyes. They speculated that contraction of the retina secondary to stretching by the epiretinal membrane was responsible for the decrease in the YCA and deterioration in visual function. There was no significant difference in the retinal artery angle between the mild and severe phenotype groups in our study. The small sample size may account for the absence of detectable differences, and retinal folds and shrinkage were common in several severely affected eyes. This made it difficult to identify and measure certain regions, such as the optic disc and macula. We also found that the incidence of foveal hypoplasia was high in FEVR eyes, especially in those with severe disease. Chen et al. [29] reported that hypoplasia of the inner retinal layer of the fovea occurred in 48.78% of FEVR eyes, which supports our findings. Foveal hypoplasia seemed to be related with angiodysplasia, including a small retina artery angle, small FAZ area and lower FD-300, SCP and DCP. When the FAZ is completely surrounded by parafoveal capillary beds, the cells of the inner retina can be pushed centrifugally to form the foveal pit during the maturation of the fovea [30]. In our study, the vascular density of the parafoveal layer and FD-300 were lower in eyes with FEVR. We postulate that this mechanism was impaired in some patients with FEVR, leading to foveal hypoplasia. These results suggest the presence of pathological changes in the fovea in addition to the peripheral retina. Moreover, Zhang et al. [31] reported that the decrease in the vascular density of the SCP was independently related to the severity of FEVR and vision loss. These findings indicate the potential correlation between vascular density and fovea development.

The relationship between the genotype and clinical phenotype of FEVR has been frequently studied and discussed in the literature. Our results showed that the vascular density of the DCP in the whole and parafoveal images was smaller in patients with digenic inheritance of FEVR than that in patients with monogenic inheritance. Li et al. [32] reported that most eyes of 13 probands with two disease-causing variants had stage 4 (38.46%) or stage 5 (26.92%) disease. A case series detected a heterozygous biallelic variant in *FZD4* leading to hearing deficits and developmental delays [19]. In *LRP5* variants, a higher incidence of severe phenotype was observed in patients with biallelic variants compared to those with monoallelic variants [33]. These finding may be related to the cumulative effect of the multiple and complex signal pathways related to FEVR.

We determined that FEVR of greater severity was more frequently associated with *FZD4* compared to *LRP5*. Foveal hypoplasia was noted in 55% patients with *FZD4* variants. Chen et al. [29] searched for variants in 27 FEVR-afflicted families and reported that a preserved foveal IRL or small FAZ area did not occur in patients with FEVR with the *LRP5* variants, implying that *LRP5* causes mild phenotypic manifestations, consistent with our results. However, Seo et al. [34] found that an *FZD4* variant seemed to result in a milder phenotype compared to *LRP5,* based on the comparison of FEVR severity and visual acuity in 18 patients. A study including 89 patients with unilateral or bilateral retinal folds found that the former were observed in 87.5% (14/16) and 73.7% (14/19) of patients with *LRP5* and *FZD4* variants, respectively, suggesting that patients with *LRP5* and *FZD4* variants manifested milder phenotypes and a higher frequency of asymmetry [35]. Binocular involvement and severe manifestation occurred in 79.2% (38/48) patients with NDP variants [36]. The proband with NDP in this study also exhibited binocular involvement and underwent surgery. The phenotype in KIF11 was different from other FEVR genes. The retinopathy in patients with FEVR presented as chorioretinal dysplasia [37]. The AI of the FAZ obtained from OCTA imaging was significantly lower in *TSPAN12* eyes than that in eyes with the *LRP5* variants. The foveal vascular density in the SCP and DCP was lower in FEVR patients with *LRP5* than that in patients harbouring *TSPAN12* variants. FD-300 was also lower in eyes with *LRP5* variants compared to eyes with *FZD4* and *TSPAN12* variants. Thus, we may infer that patients with FEVR with *LRP5* variants exhibited a broader phenotypic spectrum. The variations in the results of different studies may be attributed to the differences in their sample populations. Additionally, environmental factors may influence the phenotypic manifestation for each FEVR variant, and/or there may be epigenetic changes occurring during development or in the process of incomplete gene penetrance [38]. Further studies are warranted to investigate this aspect in detail.

There are some limitations to our study. First, we enrolled patients with FEVR, including probands and all their family members to investigate the clinical characteristics and genotype–phenotype relationships. This strategy could have introduced selection bias into the results. Second, the number of patients with the severe phenotype was relatively small, resulting in an uneven distribution of FEVR grades. Third, some children were too young to undergo examinations that required compliance; thus, the possibility exists that the missing data skewed the results and data analysis. These limitations are expected to be addressed in future research by enrolling a larger sample population.

## Conclusions

In summary, we discovered a total of 21 FEVR gene variants, 10 of which have never been reported. *LRP5* had the highest proportion of variants. We also reported the retinal artery angle, occurrence of foveal hypoplasia and OCTA features in FEVR, and that the presence of *FZD4* variants may lead to more severe FEVR than *LRP5* variants. In light of these findings, we suspect that other unknown genes may also cause and/or contribute to the FEVR phenotype that remain to be identified, necessitating future studies that include detailed genetic and clinical research.

## Data Availability

Data are available from the corresponding author upon reasonable request.
